# Speechreading in hearing children can be improved by training

**DOI:** 10.1111/desc.13124

**Published:** 2021-06-01

**Authors:** Elizabeth Buchanan-Worster, Charles Hulme, Rachel Dennan, Mairéad MacSweeney

**Affiliations:** 1 Institute of Cognitive Neuroscience, University College London, London, UK; 2 Deafness, Cognition and Language Research Centre, University College London, London, UK; 3 Department of Education, Oxford University, Oxford, Oxfordshire, UK

**Keywords:** hearing, phonological awareness, reading, speechreading, training

## Abstract

Visual information conveyed by a speaking face aids speech perception. In addition, children’s ability to comprehend visual-only speech (speechreading ability) is related to phonological awareness and reading skills in both deaf and hearing children. We tested whether training speechreading would improve speechreading, phoneme blending, and reading ability in hearing children. Ninety-two hearing 4- to 5-year-old children were randomised into two groups: business-as-usual controls, and an intervention group, who completed three weeks of computerised speechreading training. The intervention group showed greater improvements in speechreading than the control group at post-test both immediately after training and 3 months later. This was the case for both trained and untrained words. There were no group effects on the phonological awareness or single-word reading tasks, although those with the lowest phoneme blending scores did show greater improvements in blending as a result of training. The improvement in speechreading in hearing children following brief training is encouraging. The results are also important in suggesting a hypothesis for future investigation: that a focus on visual speech information may contribute to phonological skills, not only in deaf children but also in hearing children who are at risk of reading difficulties. A video abstract of this article can be viewed at https://www.youtube.com/watch?v=bBdpliGkbkY.

## 1 Introduction

Phonological awareness is well-established as an important predictor of reading development in hearing children (Caravolas et al., 2012; Castles & Coltheart, 2004; Hatcher et al., 2004; Hulme et al., 2002). Children with poorer phonological awareness, such as those with developmental dyslexia, tend to have difficulties with reading (Leonard, 2014; Snowling, 2000). Given the importance of phonological awareness in hearing children’s reading development it is of interest to understand how phonological awareness develops.

Phonological awareness tasks require children to manipulate the sublexical structure of words. Such tasks are believed to relate to reading because they assess the quality and specificity of abstract representations of the phonological structure of spoken words. Typically, these underlying phonological representations are thought of as depending on interactions between auditory speech input and articulatory outputs. However, information about speech is also conveyed via vision; particularly information about the place of articulation of sounds. Visual speech that is congruent with auditory speech enhances speech perception in adults (Lusk & Mitchel, 2016; Mitchel & Weiss, 2014; Sumby & Pollack, 1954) and children with and without developmental language disorder (Knowland et al., 2016). Conversely, incongruent visual and auditory speech information can disrupt speech perception. Specifically, presenting a visual /ga/ and an auditory /ba/ results in the perception of a /da/, which is not presented in either modality but is rather a combination of the two inputs (McGurk & MacDonald, 1976). Visual speech is a rich source of phonetic information (Bernstein et al., 2000; Buchwald et al., 2009; Sell & Kaschak, 2009; Soto-Faraco et al., 2007), despite the proportion of visually indistinct speech sounds (Auer et al., 1997)

In hearing infants, speech perception abilities have been shown to relate to broader language skills. Visual speech perception influences infants’ word-form recognition (Weatherhead & White, 2017) and some studies have shown a relationship between visual speech perception and concurrent and later vocabulary skills in infants and young children (Altvater-Mackensen & Grossmann, 2015; Jerger et al., 2018). Visual speech perception is not only related to vocabulary in hearing children but is also used to access phonetic information. For example, Jerger et al. (2018) showed that having visual speech information present improved children’s (aged 4–14) ability to identify and discriminate phonemes in words. Given this, it is perhaps not surprising that some studies have shown a relationship between speechreading skills and phonological awareness in hearing children with and without developmental language disorder (Heikkila et al., 2017) and in hearing adults with developmental dyslexia (Mohammed et al., 2006). In addition, audio-visual training with word-to-picture matching, but not auditory-only training, improved non-word-repetition skills in children with developmental language disorder (Heikkila et al., 2018). These studies suggest that visual speech perception may contribute to the development of multimodal phonological representations.

Given the link between visual speech perception and phonological awareness, it is perhaps also not surprising that speechreading ability relates to reading ability in hearing children (Heikkila et al., 2017; Kyle et al., 2016; Kyle & Harris, 2011) as well as deaf children (Harris et al., 2017; Kyle & Harris, 2010; Kyle & Harris, 2011; Kyle et al., 2016). In addition, we have previously found that the relationship between concurrent measures of speechreading and single-word reading is mediated by phonological awareness in young hearing children as well as in deaf children (Buchanan-Worster et al., 2020). Improving speechreading ability may improve hearing children’s access to the phonetic information in speech, allowing them to build more robust multimodal phonological representations, which may in turn support reading in those with poor reading skills.

To investigate whether visual speech information contributes to the development of phonological awareness skills in hearing children, it is important to first establish whether speechreading skills can be improved in hearing children. Some researchers have claimed that speechreading is a “hard-wired” skill that cannot be trained (e.g., Summerfield, 1992). However, many studies have shown modest improvements in speechreading skill in deaf and hearing adults following training (Bernstein et al., 2001; Blumsack et al., 2007; Bothe, 2007; Lonka, 1995; Walden et al., 1977). In deaf children, we have recently shown that speechreading training using the Speechreading Training and Reading (STAR) programme led to improvements in sentence-level speechreading and in speech production, which was used as a proxy measure of their phonological representations (Pimperton et al., 2019). However, no improvement was seen on explicit measures of phonological awareness (onset- and rime-matching tasks). One reason for this may be that the phonological awareness assessment used was a multiple-choice task, making this a less sensitive measure than a free-response task.

The primary goal of the current study was to assess whether speechreading can be trained in hearing children. A secondary question was whether such speechreading training, including visual-only phoneme blending games, would also lead to improvement on phonological awareness tasks. The STAR training programme was adapted for use with 4-5-year-old hearing children based on the deaf children’s performance in the Pimperton et al. (2019) study and feedback from teachers involved in the study.

We recruited 92 hearing children aged 4-5 years old. Half the children (training group) completed three weeks of speechreading training games and the other group acted as business-as-usual (BAU) controls. Before and after training both groups were tested on measures of speechreading, phonological awareness, and single-word reading, which were comprised of a set of words used in the training and a matched set of untrained words.

For each of the single word speechreading, phonological awareness, and single-word reading tasks we predicted that the speechreading trained group would perform better than the control group on post-tests when controlling for baseline scores on that test and that this would generalise to untrained words as well as trained words. We also predicted that the speechreading trained group would perform better than the control group on a measure of sentence speechreading involving unseen models and stimuli.

## 2 Method

### 2.1 Design

The design for this study was pre-registered on the Open Science Framework (pre-registration: https://osf.io/wyc84, modified analysis plan: https://osf.io/8kh9m). Ethical approval for the study was granted by the UCL Research Ethics Committee (3612/006) and informed consent was provided by parents for the participating children. This study was a randomised experiment with hearing children aged 4 to 5 years old. Children were assessed at pre-test (T1) on measures of speechreading, phonological awareness, and single-word reading. Pre-test assessments took place in the week before the intervention commenced. Children were then randomised to the speechreading intervention group or to a BAU control group. All children were then re-assessed again in the week immediately after the 3-week intervention period (T2). After the T2 assessment it was decided to follow-up the children after 3 months (T3) to assess whether the intervention effects were maintained. The T3 assessments were not included in the pre-registration (https://osf.io/wyc84). Therefore, the T3 analyses are reported here as exploratory. The intervention was run by the experimenter and therefore was not blinded.

### 2.2 Participants

**Figure 1:**
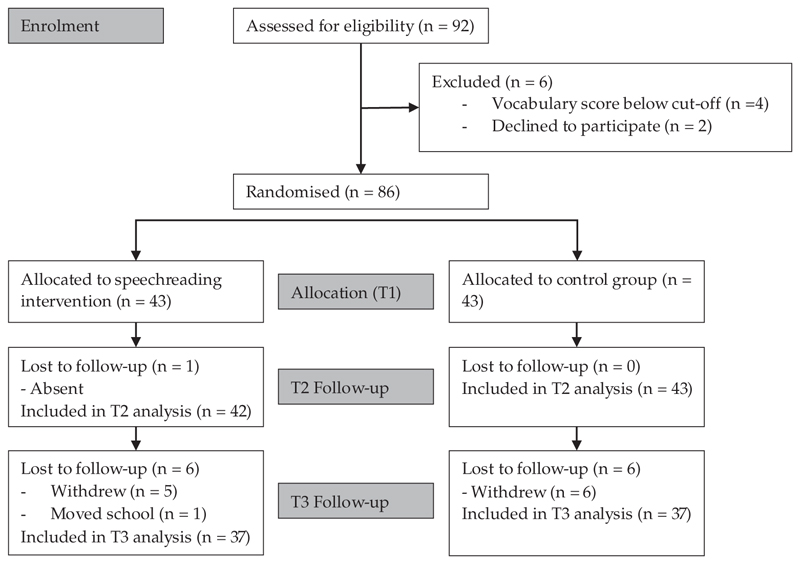
Flowchart documenting the movement of participants through the different stages of the trial

Ninety-two 4-to-5-year-old hearing children were recruited from five mainstream schools (seven classes) in Cambridgeshire and London. The CONSORT diagram in Figure 1 shows the flow of participants through the trial. Two children were excluded because they were unresponsive in baseline tasks and four were excluded because they had a vocabulary score more than two standard deviations below the mean of the group (*M* = 37.26, *SD* = 4.73, cut-off was 27.80). The remaining 86 children (38 females) had a mean age of 4 years and 11 months (SD = 3.7 months; range: 52–65 months) at baseline. The children participated in this study in the second term of their first year at school. Pilot work indicated that at the beginning of their first term of the first year at school children were not able to complete any of the assessment tasks but children in the first term of Year 1 (second year at school) were already at ceiling on many of the phonological awareness tasks. By the end of their first term at school, pilot children were able to attempt most of the tasks. Therefore, conducting the intervention in the second term of school was considered appropriate.

The children were randomised using stratified randomisation within classes and schools in Stata (Version 15.1; StataCorp. 2017). There were 43 children (18 female) in the intervention group and 43 (20 female) in the BAU control group. There were no differences between the groups in age (*t*(84) = 0.06, *p* = 0.953, *d* = 0.01) or performance on any of the three phonological awareness tasks at baseline (Syllable blending: *t*(84) = 1.25, *p* = 0.217, *d* = .27; Phoneme Blending: *t*(84) = –0.31, *p* = 0.754, *d* = –0.07; Phoneme Deletion: *t*(84) = –0.36, *p* = 0.721, *d* = –0.08).

### 2.3 Assessments

All children were assessed at baseline (T1), immediately after the intervention time (T2) and 3 months after the intervention (T3) on vocabulary, speechreading, phonological awareness, and single-word reading measures. All these measures included 20 words from the intervention (trained words) and the matched list of untrained words. Including 20 words in each list provided a focused set of training words whilst maintaining a sufficient number of training words to avoid excessive repetition. The trained and untrained items were alternated trial by trial throughout the assessment tasks.

#### 2.3.1 Stimuli

Two lists of 20 words were compiled from a total of 103 words from Pimperton et al. (2019). One list of words were trained, the other was not. The lists were matched for the average number of phonemes, letters, and syllables; frequency (KF: Kucera & Francis, 1967—count of words from a database of just over a million words); the visibility of the words (the proportion of hearing adults who were able to speechread the words, Pimperton et al., 2017) and the name agreement of the pictures (data from young hearing children, Pimperton et al., 2019), as shown in Table 1. One list was then selected as the training set based on the words in that list being spread across different levels of the STAR game (trained words).

#### 2.3.2 Vocabulary

The vocabulary task consisted of images of all 40 items from the trained and untrained sets. Children were asked to name each item individually. If a child provided a similar label (e.g., “bunny” instead of “rabbit”) they were prompted to provide a different label (“Can you think of another word for that?”). If the child could not name an item, the correct label was provided. The vocabulary task was only used at pre-test as a screening measure to ensure the children had a suitable proficiency in English. Those who scored less than two standard deviations below the mean on the vocabulary measure at pre-test were not further included in the trial. All remaining children had a minimum score of 31/40 on the vocabulary task.

#### 2.3.3 Speechreading

##### Single word speechreading—Primary outcome measure

The single word speechreading task was made up of silent videos of four models speaking all 40 items. The videos were ordered in terms of ease of speechreading (Pimperton et al. 2019), with the easiest words to speechread presented first. The trials alternated between trained and untrained items. The videos included the four models from the training game and the order of models was randomised. There were three practice trials at the start and after each of these the child was given verbal and visual feedback, with the correct answer circled on the screen. The first 20 trials were multiple-choice. After each video was played four images were presented at the four corners of the screen, including the target item and three unrelated images. Each child was asked to point to the image they thought matched the word in the video. The second half of the task was free-response. Each child was asked to say out loud what they thought the person had said in the video. They scored a point for each item correctly identified throughout the task, making a total of 40 points. If a child got 5 items incorrect in a row, the task was stopped.

##### Sentence speechreading

Sentence speechreading was assessed with the “Everyday Questions” subtest of the Test of Child Speechreading (Kyle et al., 2013, https://dcalportal.org/). In this task, the children watched 12 silent videos of a speaker (six male, six female) asking an everyday question, such as “How old are you?”. After each video the child was asked to repeat the question and were awarded one point for each word correctly identified, regardless of word order. Children could score a total of 62 points. For full details see Supplementary Materials of Pimperton et al. (2019, https://doi.org/10.23641/asha.8856356). This task was not included in the exploratory 3-month follow-up (T3) in order to reduce testing time.

#### 2.3.4 Phonological awareness

Phonological awareness was assessed at three levels: syllable blending, phoneme blending, and phoneme deletion. The 40 trained and untrained items were divided across these three tasks as described below.

##### Blending

The blending tasks were modelled on the Clinical Evaluation of Language Fundamentals four (CELF 4; Semel, Wiig, & Secord, 2003) tasks, using the same instructions and practice items. At the beginning of the tasks the experimenter said “I will say a word very slowly. I want you to tell me what I’m saying”. The experimenter then said either each syllable or each phoneme in the word. The syllable-blending task included six items (three trained), for example “snowman” presented as “snow— man”.The phoneme blending task included 14 items (seven trained), for example "boot" presented as "/b/ /oo/ /t/". Experimenters were trained to break down the words and present units (syllable or phoneme) at approximately 1 per second. The blending tasks were not included in the exploratory 3-month follow-up (T3) as many children had already reached ceiling on these tasks by the immediate post-test (T2).

##### Phoneme deletion

The sound deletion task was modelled on theYARC sound deletion task (Hulme et al., 2009), using the same instructions and practice items. It included deletion of syllables (e.g., “icecream” without “cream”), initial phonemes (e.g., “shoe” without the /sh/), final phonemes (e.g., “leaf” without the /f/) and phonemes from consonant clusters (e.g. “spoon” without the /p/). There were seven practice items plus 20 test items (10 trained). Feedback was only given on the practice items and one point was awarded for each test item. The phoneme deletion and vocabulary tasks were combined so that the child was shown an image, asked to name it and then asked to repeat the name without a specific sound. If the child got the vocabulary item wrong, they were provided with the correct answer before being asked to manipulate the sound.

#### 2.3.5 Single-word reading

In the reading task, each child was presented with the 40 words (20 trained and 20 untrained), shown on the screen four at a time. The words were ordered based on orthographic complexity and grapheme-phoneme correspondence and the trials alternated between trained and untrained items. Each child was asked to work through the screens reading the words aloud. No feedback was given. If five consecutive words were read incorrectly the task was stopped.

### 2.4 Intervention

**Figure 2:**
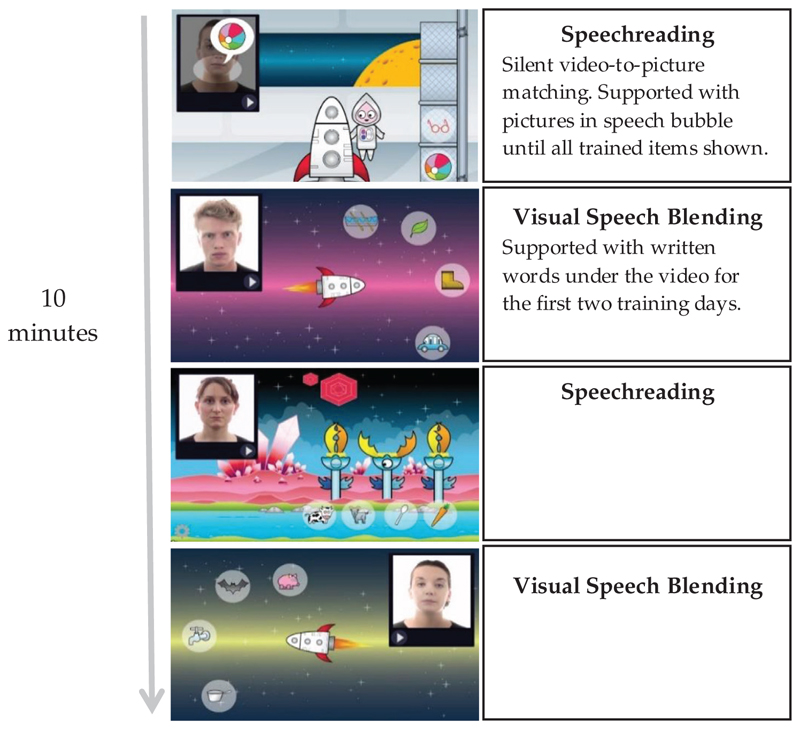
Schematic of each 10-minute training session. The first and third game of each day involved single word speechreading and selecting a matching image. The second and fourth games of each day involved blending silent videos of the model breaking down a word (e.g., “b-a-t”) and selecting the matching picture. The planet that the children “visited” changed each day (four different planets) in order to maintain interest.

The speechreading training game was adapted from Pimperton et al. (2019). Each session followed the structure outlined in Figure 2 with further details provided in the Supplementary Materials. One aim of the current study was to assess the contribution of visual speech information to the development of phonological awareness and thus it was considered appropriate to prioritise the phonological awareness aspect of the game. Therefore, each silent 10-minute session consisted of two speechreading games and two visual speech blending games, in order to explicitly teach blending through the visual modality. The visual speech blending games were adapted from Pimperton et al. (2019) to include the trained items but not the untrained items. Blending requires the children to access phonemes by speechreading, blend the phonemes together, and match the resulting word to a picture. Blending was selected as it is widely taught as an important element of auditory phonological awareness in the context of phonic reading instruction.

The children played the games in a room with 1–4 other players at a time, each playing on their own touch-screen device (Microsoft-Surface Pro) via the internet. These sessions were supervised by the experimenter. In every game the trials only progressed once the child had selected the correct response. If a child responded incorrectly they had to re-watch the video before being able to select any of the response options. The games progressed using an adaptive algorithm based on each child’s performance. The games became progressively more difficult in two ways: (1) by changing the level of support (text on the screen) and (2) the similarity of the distractors to the target. These features are further detailed in the Supplementary Materials.

### 2.5 Business as usual control group

The BAU control group were seen by the experimenter at pre- and post-test only. As children in the intervention group participated at different times during the school day, the BAU control group were doing a variety of school activities during the intervention period. All children (intervention and control) received 30 min of phonic reading instruction in school every day in line with the National Curriculum in the UK. The BAU control group did not receive any additional intervention. Children in Reception (aged 4/5 years) receive daily phonics lessons, which include recognition of individual letters and digraphs and blending of words. The classes aim to prepare the children to be able to decode words by the end of year 1 (aged 6), which is assessed by the phonics screening check involving reading 20 words and 20 pseu-dowords (https://www.gov.uk/education/phonics).

### 2.6 Statistical methods

Differences in performance between the speechreading intervention group and the control group at T2 and T3 were assessed using ANCOVAs. The dependent variable in each model was performance on the assessment at either T2 or T3, the covariate was performance on that assessment at T1 and the fixed factor was the group (intervention or control). Each ANCOVA was run three times: once with both trained and untrained words included, once with only trained words, and once with untrained words. Performance on each of the measures was considered to be normally distributed based on visual inspection of Q-Q plots. The assumption of equality of slopes was assessed by including an interaction term in the ANCOVA models between the covariate and the group.

## 3 Results

Assessment data were collected from 86 children at T1 (43 speechreading intervention, 43 control), 85 children at T2 (42 speechreading intervention, 43 control), and 74 children at T3 (37 speechreading intervention, 37 control) (see Figure 1—CONSORT diagram). Adherence to the intervention was high because the intervention was implemented directly by the research team. Forty-two of the 43 children in the intervention group completed all 15 training sessions. One child completed 14 of the 15 training sessions.

Table 2 shows the means, standard deviations and Cohen’s *d* for each group at baseline (T1), immediate post-test (T2) and 3-month follow-up (T3). Table 3 shows these descriptive statistics for the trained and untrained words separately. Cohen’s *d* was calculated as the difference in gains between groups from T1 to each post-test divided by the pooled standard deviation at T1.

### 3.1 Planned analyses—effects of training at T2

#### 3.1.1 Single word speechreading

Separate ANCOVAs of T2 single word speechreading scores, controlling for baseline single word speechreading scores, showed an advantage for the intervention group over the BAU control group for all words (trained and untrained combined: difference in marginal means = 3.88 [95% CI 1.74, 6.03]; *t* = 3.60, *p <* 0.001); trained words (difference in marginal means = 2.64 [95% CI 1.33, 3.95]; *t* = 4.01, *p <* 0.001) and untrained words (difference in marginal means = 1.28 [95% CI 0.23, 2.32]; *t* = 2.43, *p* = 0.017).

#### 3.1.2 Sentence speechreading

An ANCOVA of T2 TOCS Extension scores, controlling for baseline TOCS extension scores, showed no significant difference between the speechreading intervention and BAU control groups (marginal mean difference = 0.51 [95% CI –2.14, 3.16]; *t* = 0.381, *p* = 0.704).

#### 3.1.3 Phonological awareness

The pre-registration specified that performance on the three phonological awareness tasks would be combined if they showed a strong correlation (*r* > .60) with each other. Syllable blending was excluded as a measure due to ceiling effects (see Table 2). Phoneme blending and phoneme deletion scores did not correlate with each other to the pre-specified level (T1: *r* = .53; T2: *r* = .54) and so were therefore not combined. The planned analyses (ANCOVA and t-tests) are therefore reported for each measure independently below.

##### Phoneme blending

Separate ANCOVAs of T2 phoneme blending scores, controlling for baseline phoneme blending scores, showed no significant difference between the speechreading intervention and BAU control groups on all words (trained and untrained combined: marginal mean difference = 0.21 [95% CI –0.34, 0.77]; *t* = 0.76, *p* = 0.449); trained words (difference in marginal means = 0.32 [95% CI –0.04, 0.68]; *t* = 1.77, *p* = 0.081) and untrained words (difference in marginal means = –0.10 [95% CI –0.51, 0.32]; *t* = –0.46, *p* = 0.645).

**Figure 3:**
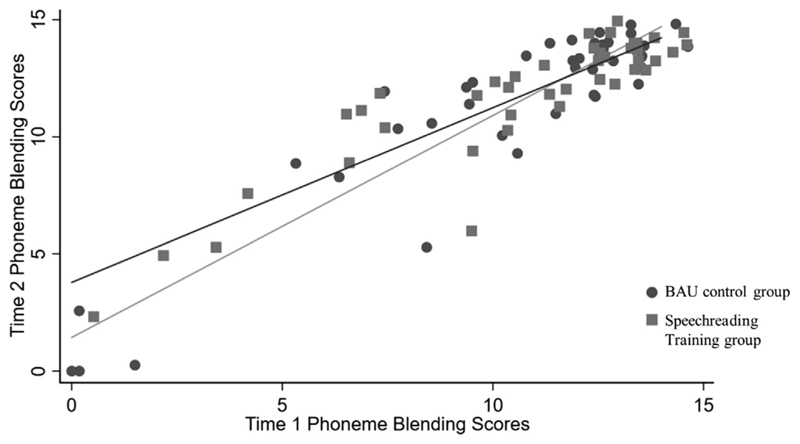
Phoneme blending scores at T1 and T2, showing an interaction of the slopes for the two groups. The circles and pale grey line show data for the BAU control group. The squares and dark grey line show data for the intervention group.

The assumption of equal slopes was not met for the phoneme blending task (trained and untrained words combined), the interaction term between the group and covariate was significant (unstandardised slope = –0.20 [95% CI –.35, –.06]) as shown in Figure 3. This was also true when analysing the trained items only (unstandardised slope = –0.23 [95% CI –.42, –.05]). This corresponds to a shallower slope for the intervention group, meaning that the intervention led to a greater improvement in phoneme blending for children who initially had lower scores on this measure. Exploratory follow-up tests on trained and untrained items combined indicated that the groups did not differ at post-test at the mean of the covariate (marginal mean difference = 0.22 [95% CI –.32, .75]; *t* = 0.80, *p = 0.426).* However, for children scoring 1 standard deviation below the mean at pre-test (the covariate, *n* = 10) there was a significant advantage for the intervention group at post-test (marginal mean difference = 0.97 [95% CI .21, 1.74]; *t* = 2.53, *p* = 0.013). These results must be interpreted with caution as there were few children scoring at the lower end on this measure. However, this pattern suggests that the speechreading training was effective for children with low initial phoneme blending scores.

##### Phoneme deletion

Separate ANCOVAs of T2 phoneme deletion scores, controlling for baseline phoneme deletion scores, showed no significant difference between the speechreading intervention and BAU control groups on all words (trained and untrained combined: marginal mean difference = 0.39 [95% CI –0.89, 1.68]; *t* = 0.605, *p* = 0.547); trained words (difference in marginal means = 0.23 [95% CI –0.50, 0.96]; *t* = 0.63, *p* = 0.530) and untrained words (difference in marginal means = 0.18 [95% CI –0.55, 0.91]; *t* = 0.49, *p* = 0.627).

#### 3.1.4 Single-word reading

Separate ANCOVAs of T2 single-word reading scores, controlling for baseline single-word reading scores, showed no significant difference between the speechreading intervention and BAU control groups on all words (trained and untrained combined: marginal mean difference = –0.69 [95% CI –2.54, 1.15]; *t* = –0.75, *p* = 0.456); trained words (difference in marginal means = 0.23 [95% CI –0.85, 1.31]; *t* = 0.42, *p* = 0.672) and untrained words (difference in marginal means = –0.87 [95% CI –1.82, 0.07]; *t* = –1.84, *p* = 0.069).

### 3.2 Exploratory analyses—3-month follow-up (T3)

Given the improvement on speechreading scores for the intervention group over the control group at T2, the children were followed up 3 months post intervention (T3) to determine whether the improvement on speechreading skill was maintained and whether there was a knock-on effect on phonological awareness and single-word reading ability. The analyses reported at T2 were repeated with T1 and T3 scores.

#### 3.2.1 Speechreading

Separate ANCOVAS of T3 single word speechreading scores, controlling for baseline single word speechreading scores, showed an advantage for the intervention group over the BAU control group for all words (trained and untrained combined: (marginal mean difference = 2.46 [95% CI 0.42, 4.50]; *t* = 2.40, *p* = 0.019); trained words (difference in marginal means = 1.25 [95% CI 0.18, 2.31]; *t* = 2.33, *p* = 0.023) and untrained words (difference in marginal means = 1.23 [95% CI 0.08, 2.37]; *t* = 2.13, *p* = 0.036).

#### 3.2.2 Phoneme deletion

Separate ANCOVAS of T3 phoneme deletion scores, controlling for baseline phoneme deletion score, showed no significant difference between the speechreading intervention and BAU control groups for all words (trained and untrained combined: marginal mean difference = 0.07 [95% CI –1.38, 1.52]; *t* = 0.10, *p* = 0.923) trained words (difference in marginal means = –0.22 [95% CI –1.06, 0.62]; *t* = –0.518, *p* = 0.606) or untrained words (difference in marginal means = 0.31 [95% CI –0.52, 1.13]; *t* = 0.735, *p* = 0.465).

#### 3.2.3 Single-word reading

Separate ANCOVAS of T3 single-word reading scores, controlling for baseline single-word reading score, showed no significant difference between the speechreading intervention and BAU control groups (trained and untrained combined: marginal mean difference = 0.14 [95% CI -3.25, 3.53]; *t* = 0.08, *p* = 0.934); trained words (difference in marginal means = 0.88 [95% CI -0.85, 2.62]; *t* = 1.014, *p* = 0.314) or untrained words (difference in marginal means = -0.70 [95% CI -2.57, 1.16]; *t* = -0.750, *p* = 0.456).

## 4 Discussion

The current randomised experiment investigated whether speechreading can be trained over 3 weeks in young hearing children (4-5 year olds) and whether this training transfers to improvements in phonological awareness.

### 4.1 Speechreading

As predicted, speechreading training led to improvements in speechreading performance in hearing 4–5 year old children. The speechreading intervention group performed better than the BAU control group on the single word speechreading post-test when controlling for baseline scores on the same test. In addition, the speechreading intervention group not only improved on trained words but also on untrained words as a result of the intervention. These effects were maintained at the 3-month follow-up.

These results challenge the idea that speechreading is a fixed skill that cannot be trained (Montgomery & Sylvester, 1984; Summerfield, 1992). There is evidence that speechreading can be trained in deaf (Bothe, 2007; Lonka, 1995; Walden et al., 1977) and hearing adults (Bernstein et al., 2001; Blumsack et al., 2007). The Speechreading Training and Reading (STAR) programme has been shown to be effective in improving speechreading in deaf children Pimperton et al. (2019). The current study furthers this literature showing that speechreading can also be trained in hearing children. This suggests that improvements in speechreading in young children can arise as a result of short-term training regardless of the extent to which the individual relies on visual speech information to access spoken language in daily life.

Although improvements were seen on the single word speechreading task, the speechreading training did not result in improved performance on the Test of Child Speechreading (TOCS) Everyday Questions extension task (repetition of everyday questions). This may be due to the differences between the tasks. Unlike the TOCS extension everyday questions task, the single word speechreading task had a similar format to the training games, involving the same talkers, multiple-choice responses, and single-word stimuli. First, it is possible that the very brief training would not extend to untrained talkers (Lander & Davies, 2008). Second, it may be that even if the children improved in identifying phonemes, sufficient to allow them to select options in the multiple-choice single word speechreading task, this may not have been sufficient to observe improvements in the free-response everyday questions task. Finally, the sentence-level stimuli in the everyday questions task may have been too linguistically complex for these young children. Sentence-level speechreading ability is generally worse than single word speechreading ability in deaf and hearing adults and children (Kyle et al.,2013; Mohammed et al., 2006). In addition, despite being reminded they were to repeat a question, many children provided a statement response or a single word indicating that they may not have understood the linguistic structure of a “question” when this meta-linguistic knowledge was tested explicitly.

The improvements seen on single word speechreading but not on sentence-level speechreading (everyday questions test) as a result of the intervention in hearing children are in contrast to the assessment of the STAR programme with deaf children, who showed the opposite pattern (STAR_D, Pimperton et al., 2019). Both the hearing children in the current study and deaf children in the STAR_D study improved on single word speechreading tasks with familiar talkers and trained items (see “in-game” assessments, Pimperton et al., 2019). However, the hearing children in the current study also improved on speechreading untrained items with familiar talkers whereas the deaf children did not (see “in-game” assessments). The deaf children also did not improve on a speechreading task with unfamiliar talkers and stimuli (TOCS single word speechreading test). The hearing children in the current study did not improve on the TOCS Everyday Questions extension task whereas the deaf children in the STAR_D study did.

One reason for the difference in results between the current study and the STAR_D project may be that the training was over three times longer in the STAR_D project (12 weeks, 48 sessions) than in the current study (3 weeks, 15 sessions). In addition, the children in the STAR_D training played some games that used sentences, for example “Find the sheep.”. It may be that the longer period of training and the exposure to sentences in the training is necessary to lead to improvements in the more complex sentence-level speechreading as tested in the TOCS Everyday Questions extension task.

### 4.2 Phonological awareness

Having shown improvement in speechreading, which generalised to untrained words, following training our secondary aim was to investigate whether speechreading gains would lead to improvements in phonological awareness skills. At the group level there were no effects of speechreading training, with no differences in performance between the speechreading training group and the control group on post-tests of either phoneme blending or phoneme deletion when controlling for baseline scores. This was the case for both trained and untrained words. Follow-up analyses three months later also showed no improvement at the group level in phoneme deletion scores as a result of the intervention. However, despite no group effects on performance on phonological awareness tasks, the poorest performing children did show improvements on phoneme blending as a result of training.

At the group level, an effect on phonological awareness may not have been observed as the improvement in single word speechreading was moderate, and therefore may not have been large enough to lead to changes in phonological awareness. Perhaps more importantly, both the speechreading intervention and the BAU control groups made large improvements from baseline to both post-test timepoints on the phoneme deletion task, reflecting the fact that phonics instruction is a dominant aspect of the curriculum for this age group (Rose, 2006). The current study did not select children based on their performance on the phonological awareness tasks and therefore included many children who were already at ceiling on the phonological awareness tasks or who made rapid improvements over the course of the study. Any improvements on the phonological awareness tasks as a result of the speechreading intervention are likely to be small and therefore may not have been detected in the context of the huge gains made (range of improvement on phoneme deletion: –3–14 points). In addition, pilot work revealed that at the beginning of the school year (September) the 4 year old children could not attempt the phonological awareness tasks. However, at pre-test, after one term in school (January-March), many were already at ceiling on the phoneme blending task. This huge leap in performance in the majority of children makes it unlikely we would observe any improvement on the phoneme blending task due to the manipulation of speechreading training.

Although many children make rapid progress in phonological awareness skills in their first few months of school, many children struggle. It is likely to be specifically these children that may benefit from their attention being brought to visual speech in order to augment and support their understanding of the sublexical structure of words through sound alone (Heikkila et al., 2017; Knowland et al., 2016; Mohammed et al., 2006). The children in the intervention group who started off with low scores on the phoneme blending task performed better at post-test than the BAU control group children with low baseline scores. The observed interaction may indicate that practicing blending in the visual modality alone may lead to improvements in phonological awareness. Future studies are needed separate out the effects of the visual element of the training versus the focus on blending skills. It is also possible that small differences in the very few poorest performing children (10/85 children scored more than one standard deviation below the mean of the group at T1, see Figure 3) were exaggerated in the analysis by the clustering of children performing at ceiling on the phoneme blending task (55/85 children scored more than 10/14). For those children performing at ceiling, the task may not capture the variation in their abilities or any improvement made over time or as a result of the intervention. This may then mean that small differences in performance for the poorest performing children can exert more influence on the gradient of the regression line, resulting in a significant interaction. For the reasons outlined here, we cannot conclude from the current study that visual speech information does contribute to phonological awareness skills in young hearing children. However, the results do suggest that visual speech information may support the development of phonological skills in children with weaknesses in this area. Future studies targeting children with poor phoneme blending skills are needed to test this hypothesis directly and to determine whether the pattern we report here is a true effect.

### 4.3 Single-word reading

The speechreading training group did not differ in performance on the single-word reading post-tests from the BAU control group at the immediate follow-up or the 3-month follow-up, on either trained or untrained items. It is not surprising that there were no differences in single-word reading as a result of the intervention as single-word reading is strongly predicted by phonological awareness ability in hearing children, and, in the children as a group, this did not improve as a result of the intervention (Caravolas et al., 2012; Castles & Coltheart, 2004; Hatcher et al., 2004; Hulme et al., 2002). As with the phonological awareness tasks, these young children made huge gains in their reading skills during their first year at school. Therefore, any small improvement that may have resulted from the intervention may have been undetectable in this context.

### 4.4 Limitations

There are some features of the design that limit the interpretation of the results. The small training dosage meant that any improvements in phonological awareness as a result of the intervention were likely to be small. During the same period as the intervention many children made huge improvements in their phonological awareness skills in both groups and therefore small intervention effects would be difficult to observe. Future studies targeting those with poor phonological awareness skills would address this limitation. Another limitation of the design was that the experimenters conducting the post-tests also oversaw the training sessions. Therefore, they were not blinded to the conditions the children were assigned to, which could bias the results.

## 5 Summary

This study demonstrated the efficacy of the STAR intervention in young hearing children, showing that speechreading can be trained in hearing children as well as deaf children (Pimperton et al., 2019) even with 2.5 hours of training spread over 3 weeks (5 days a week, 10 min/day). This improvement in speechreading was not limited to words that were included in the training but generalised to untrained words. With a very short training dose it is not surprising that there were not detectable gains at the group level in phonological awareness or single-word reading, especially in the context of the 30 min of phonics training children receive each day as part of the national curriculum. Although the interaction effect observed here between group and baseline scores on the phoneme blending task suggests that speechreading may contribute to phoneme blending in those with poor phonological skills, future studies targeting children with poor phonological awareness skills are needed to test this hypothesis directly. Given the multimodal nature of speech, understanding the potential role of visual speech information in the development of phonological skills is not only relevant to education for deaf children but also for hearing children. This will ensure that children have access to all possible tools to develop their phonological skills to their best potential.

## Supplementary Material

Movie S1

Supplementary material

## Figures and Tables

**Table 1:** Characteristics of the trained and untrained words used the study

	Untrained items		Trained items	
	M (*SD*)	Range	M (*SD*)	Range
**Phonemes**	3.95 (*1.32*)	2-7	3.75 (1.33)	2-7
**Letters**	5.00(*1.41*)	3-9	4.95 (1.50)	3-9
**Syllables**	1.40 (*0.68*)	1-3	1.25 (0.55)	1-3
**Frequency (KF)** ^a^	73.72 (*149.55*)	1-591	68.39 (*102.77*)	4-431
**Speech visibility** ^b^	0.27 (*0.21*)	0.04-0.79	0.25 (*0.16*)	0.05-0.51
**Name agreement** ^c^	0.96 (*0.07*)	0.80-1	0.94 (*0.08*)	0.74-1

a Kucera & Francis (1967) count based on just over 1 million words.

b Proportion correct responses from hearing adults (Pimperton et al., 2017).

c Proportion correct responses from pilot children (Pimperton et al., 2019).

**Table 2:** Descriptive statistics for each task in the assessment battery for the intervention and BAU control groups

	Intervention		BAU control		
	M	SD	M	SD	Cohen’s *d*
Vocabulary (0−40)					
T1	38.33	1.91	37.95	2.48	
Single word speechreading (0−40)					
T1	4.83	3.59	5.02	4.34	
T2	10.05	1.40	6.30	1.78	.99
T3	7.92	5.96	6.11	4.51	.50
TOCS extension words score (0−62)					
T1	4.38	5.49	5.33	6.13	
T2	7.38	7.65	7.53	6.99	.14
Syllable blending (0−6)					
T1	4.43	2.20	4.98	1.79	
T2	5.55	1.23	5.23	1.73	.43
Phoneme blending (0−14)					
T1	10.67	3.53	10.49	4.01	
T2	11.74	2.86	11.37	4.02	.05
Phoneme deletion (0−20)					
T1	6.50	4.59	6.30	4.95	
T2	8.29	4.80	7.72	5.44	.08
T3	10.38	4.83	10.11	4.93	.01
Single-word reading (0−40)					
T1	8.81	9.95	7.16	7.79	
T2	11.79	11.84	10.65	9.63	−.06	
T3	20.05	11.81	17.53	12.36	.10

*Note*. At T1*n* = 86, at T2 *n* = 85, at T3 *n* = 74.

**Table 3:** Descriptive statistics for the assessment battery tasks for the intervention and BAU control groups for trained and untrained words separately

	Trained words					Untrained words				
	Intervention		BAU control			Intervention		BAU control		
	Mean	SD	Mean	SD	Cohen’s *d*	Mean	SD	Mean	SD	Cohen’s *d*
**Single word speechreading (0-20)**										
**T1**	2.44	2.22	2.19	2.18		2.58	1.99	2.95	2.38	
**T2**	5.55	3.86	2.79	2.78	**1.14**	4.50	2.56	3.51	2.86	**0.62**
**T3**	4.00	3.48	2.58	2.37	**0.53**	4.37	3.16	3.53	2.51	**0.55**
**Phoneme blending (0-7)**										
**T1**	5.74	1.84	5.60	2.04		5.00	2.04	4.88	2.18	
**T2**	6.29	1.29	5.88	1.93	**0.14**	5.45	1.89	5.49	2.23	**−0.07**
**Phoneme deletion (0-10)**										
**T1**	3.72	2.45	3.40	2.67	2.95	2.40	2.91	2.40	
**T2**	4.43	2.51	4.00	2.77	**0.04**	3.86	2.47	3.72	2.82	**0.04**
**T3**	5.34	2.72	5.26	2.59	**-0.10**	5.18	2.36	4.84	2.55	**0.12**
**Single-word reading (0-20)**										
**T1**	4.16	5.20	3.51	4.22		4.70	4.73	3.65	3.70	
**T2**	5.88	6.30	4.98	4.97	**0.05**	5.93	5.65	5.67	4.75	**−0.19**
**T3**	10.00	5.89	8.18	6.11	**0.25**	10.05	5.91	9.34	6.43	**−0.08**

*Note*. At T1 *n* = 86, at T2 *n* = 85, at T3 *n* = 74. Syllable blending is not included here because ceiling effects meant no comparisons were made between groups.

## Data Availability

Access to the dataset is available by emailing Dr Buchanan-Worsteron elizabeth.buchanan-worster@mrc-cbu.cam.ac.uk

## Research Highlights

- After only 3 weeks of training (10 min/day), young hearing children’s speechreading scores improved
- Speechreading improved on both trained and untrained words
- Speechreading training improved phoneme blending scores for those with initially low levels of performance on this task
- These results demonstrate that speechreading skills are malleable and may be intimately linked to the development of phonological skills in hearing, as in deaf, children
