# Supplementary material for: Speechreading in hearing children can be improved by training
Source: Dev Sci. Author manuscript; Available in PMC 2022 Jun 22. (PMC7612880; doi:10.1111/desc.13124)
Supplement: Supplementary material [file EMS145879-supplement-Supplementary_material.docx]

SUPPLEMENTARY MATERIALS

Feedback from teachers and performance from the STAR_D project were used to adapt the training games to be suitable for 4-to-5-year-old hearing children. Teachers indicated that the deaf children in the STAR_D project enjoyed the space context of the games and the points where they were able to make choices about the games (e.g. choosing a captain character to play with) and commented on 10 minutes being an appropriate length of time for daily training. Therefore, both of these elements were maintained. The teachers had also indicated that the deaf children found trials that had phrases in too hard. These were therefore not included in the current STAR_H training.

The children were trained with the speechreading training game for 10 minutes a day, 5 days a week for three weeks. This set-up was derived from Melby-Lervåg and Hulme’s (2010) study, which showed that short-term phonics training of 7 minutes a day over two weeks on blending and other phonological awareness tasks led to improvement on phoneme deletion, rhyme generation and serial recall of 10 trained words. Given that a moderate effect was seen in this study over two weeks of training, three weeks for the current study was considered an appropriate amount of time to see improvements on phonological awareness tasks for a small set of trained words.

The set-up allowed an individual child to play a maximum of two sessions a day and 8 sessions a week in order to allow missed sessions to be caught up on subsequent days. In addition to the 20 training words (included in the pre- and post-tests) a list of 10 words were included in the training games to expose the children to a range of words. The additional set was selected based on phonological similarity to training words and was presented as videos and distractor items. A further 14 words were included at higher levels of the game in order to allow for closer matching between the targets and distractors (see below for description of game progression). Words from the untrained list were never presented as videos in the games but could appear as distractor pictures.

The games progressed in two ways. First, the games progressed by changing the level of support the children received. On the first game of each day (“Pack the rocket”) a video of a model saying a single word was presented, followed by a speech bubble showing what they said. The child then selected what the model had said from two pictures. Each speechreading trial was immediately followed by a paired trial where the picture from the previous trial was presented and the child watched two videos of a model speaking and selected the word that matched the picture. Once the child had seen all the training items in this introductory format the speech bubble was removed. Blending games were included in this training as the aim was to examine the contribution of visual speech information to phonological awareness skills. Therefore, it was considered appropriate to train a phonological awareness skill, such as blending, within the speechreading training. For the blending (“Space junk”) games the children saw a model ‘sound out’ a word (e.g. the model said ‘b-a-t’) and the child had to select the matching image (e.g. of a bat). The children were supported on the blending games for the first two days of training with the word written underneath the video of the model and each letter highlighted as it was pronounced. The writing was removed on day three and only shown on the first trial of each blending game to remind the child of the task.

The second form of progression was in the type of distractors presented. The games used the algorithm from the STAR games, which allowed the difficulty level to adapt according to the child’s performance. The distractors presented were initially visually distinct from the target item and progressively increased in similarity when the child achieved a threshold for success at that level.

The visual speech blending games progressed from having the whole word spoken after the broken-down word (e.g. ‘b-a-t-bat’) to just having the broken-down word (e.g. ‘b-a-t’) when the child had been successful in identifying that word at the first level. The blending games included only the 20 trained items that were included in the pre- and post-tests. If a child completed both levels of the blending games with this set of items, the additional items were included into the blending games so they could continue playing. The same algorithm was carried across both blending games that were played each day so that progress in one game led to increased difficulty in both games.

**Supplementary Movie:** Movie 1 shows an example clip from the speechreading training games. This clip shows one of the two single word speechreading-to-picture matching games played in each 10 minute session. As shown in the clip, the videos were silent and children received visual feedback for each answer. They were required to select the correct answer before proceeding to the next trial.
